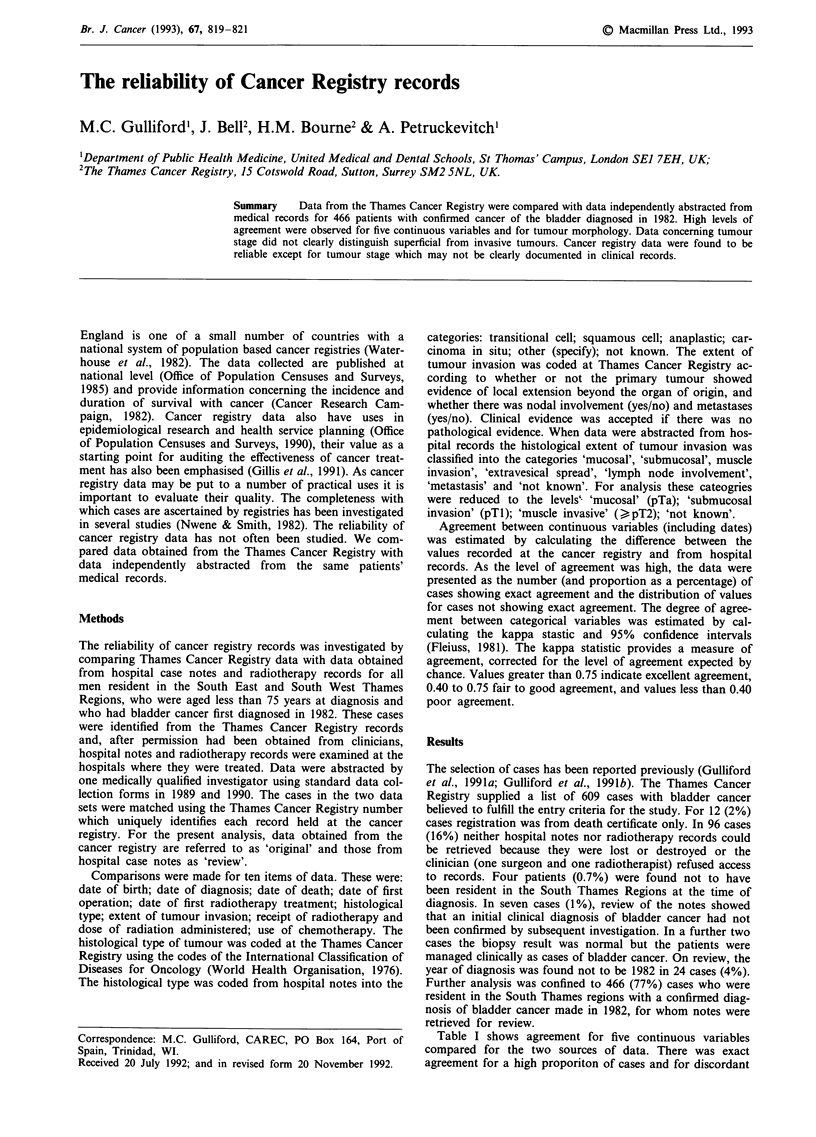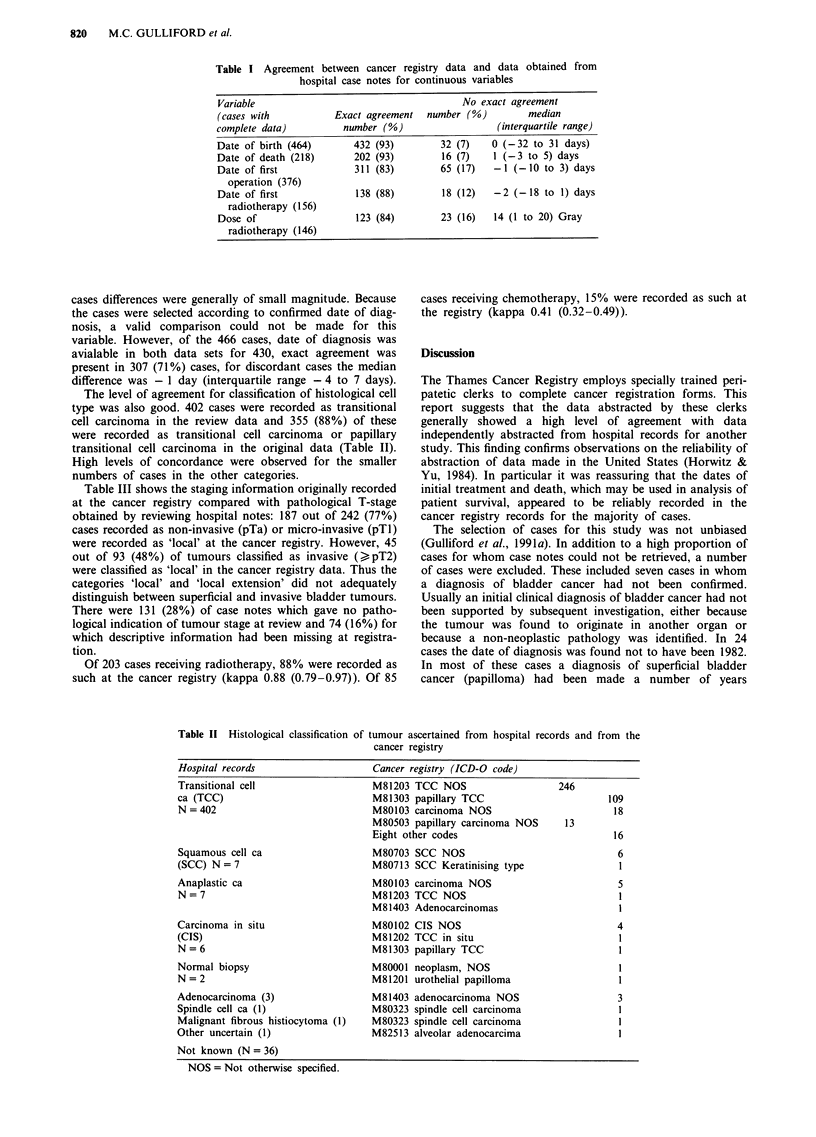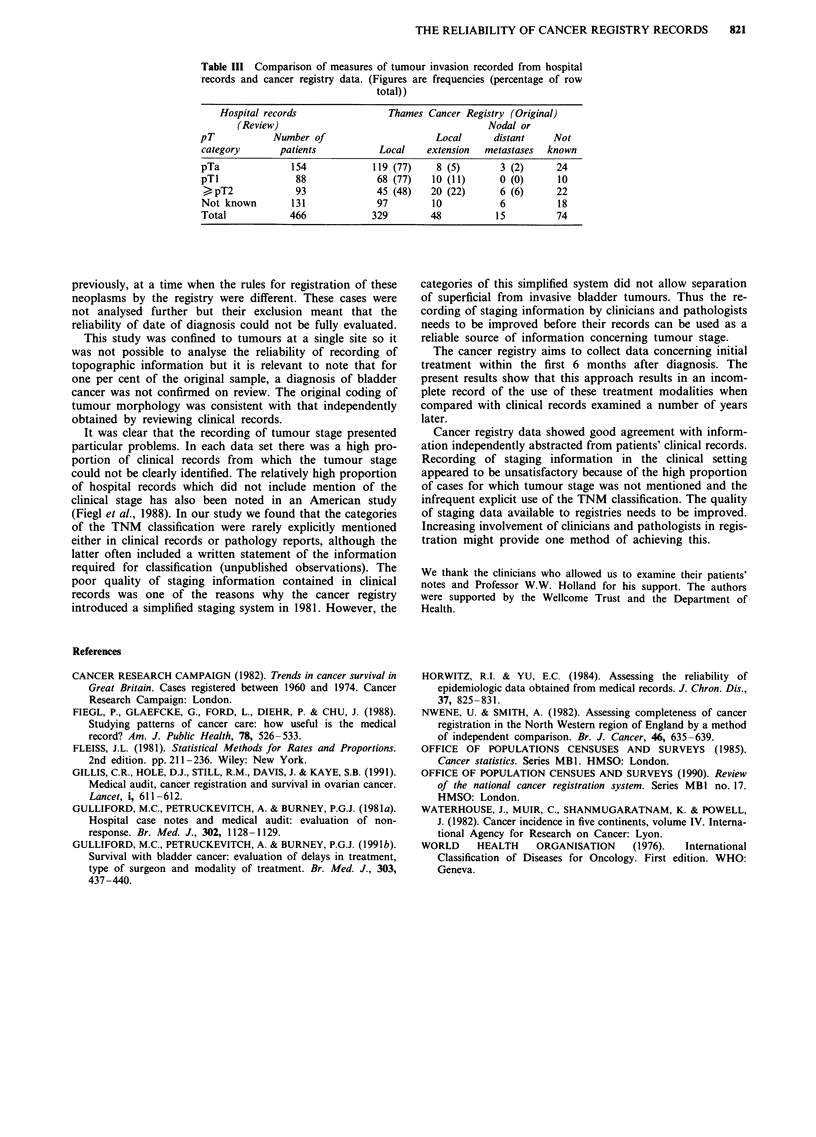# The reliability of Cancer Registry records.

**DOI:** 10.1038/bjc.1993.149

**Published:** 1993-04

**Authors:** M. C. Gulliford, J. Bell, H. M. Bourne, A. Petruckevitch

**Affiliations:** Department of Public Health Medicine, United Medical School, London, UK.

## Abstract

Data from the Thames Cancer Registry were compared with data independently abstracted from medical records for 466 patients with confirmed cancer of the bladder diagnosed in 1982. High levels of agreement were observed for five continuous variables and for tumour morphology. Data concerning tumour stage did not clearly distinguish superficial from invasive tumours. Cancer registry data were found to be reliable except for tumour stage which may not be clearly documented in clinical records.


Br. J. Cancer (1993), 67, 819-821 © Macmillan Press Ltd., 1993

### The reliability of Cancer Registry records

M.C. Gulliford[1], J. Bell[2], H.M. Bourne[2] & A. Petruckevitch[1]

[1]*Department of Public Health Medicine, United Medical and Dental Schools, St Thomas' Campus, London SE1 7EH, UK;* [2]*The Thames Cancer Registry, 15 Cotswold Road, Sutton, Surrey SM2 5NL, UK.*

**Summary** Data from the Thames Cancer Registry were compared with data independently abstracted from medical records for 466 patients with confirmed cancer of the bladder diagnosed in 1982. High levels of agreement were observed for five continuous variables and for tumour morphology. Data concerning tumour stage did not clearly distinguish superficial from invasive tumours. Cancer registry data were found to be reliable except for tumour stage which may not be clearly documented in clinical records.

England is one of a small number of countries with a national system of population based cancer registries (Waterhouse *et al.*, 1982). The data collected are published at national level (Office of Population Censuses and Surveys, 1985) and provide information concerning the incidence and duration of survival with cancer (Cancer Research Campaign, 1982). Cancer registry data also have uses in epidemiological research and health service planning (Office of Population Censuses and Surveys, 1990), their value as a starting point for auditing the effectiveness of cancer treatment has also been emphasised (Gillis *et al.*, 1991). As cancer registry data may be put to a number of practical uses it is important to evaluate their quality. The completeness with which cases are ascertained by registries has been investigated in several studies (Nwene & Smith, 1982). The reliability of cancer registry data has not often been studied. We compared data obtained from the Thames Cancer Registry with data independently abstracted from the same patients' medical records.

### Methods

The reliability of cancer registry records was investigated by comparing Thames Cancer Registry data with data obtained from hospital case notes and radiotherapy records for all men resident in the South East and South West Thames Regions, who were aged less than 75 years at diagnosis and who had bladder cancer first diagnosed in 1982. These cases were identified from the Thames Cancer Registry records and, after permission had been obtained from clinicians, hospital notes and radiotherapy records were examined at the hospitals where they were treated. Data were abstracted by one medically qualified investigator using standard data collection forms in 1989 and 1990. The cases in the two data sets were matched using the Thames Cancer Registry number which uniquely identifies each record held at the cancer registry. For the present analysis, data obtained from the cancer registry are referred to as 'original' and those from hospital case notes as 'review'.

Comparisons were made for ten items of data. These were: date of birth; date of diagnosis; date of death; date of first operation; date of first radiotherapy treatment; histological type; extent of tumour invasion; receipt of radiotherapy and dose of radiation administered; use of chemotherapy. The histological type of tumour was coded at the Thames Cancer Registry using the codes of the International Classification of Diseases for Oncology (World Health Organisation, 1976). The histological type was coded from hospital notes into the categories: transitional cell; squamous cell; anaplastic; carcinoma in situ; other (specify); not known. The extent of tumour invasion was coded at Thames Cancer Registry according to whether or not the primary tumour showed evidence of local extension beyond the organ of origin, and whether there was nodal involvement (yes/no) and metastases (yes/no). Clinical evidence was accepted if there was no pathological evidence. When data were abstracted from hospital records the histological extent of tumour invasion was classified into the categories 'mucosal', 'submucosal', muscle invasion', 'extravesical spread', 'lymph node involvement', 'metastasis' and 'not known'. For analysis these cateogries were reduced to the levels: 'mucosal' (pTa); 'submucosal invasion' (pT1); 'muscle invasive' ($\geqslant$pT2); 'not known'.

Agreement between continuous variables (including dates) was estimated by calculating the difference between the values recorded at the cancer registry and from hospital records. As the level of agreement was high, the data were presented as the number (and proportion as a percentage) of cases showing exact agreement and the distribution of values for cases not showing exact agreement. The degree of agreement between categorical variables was estimated by calculating the kappa stastic and 95% confidence intervals (Fleiuss, 1981). The kappa statistic provides a measure of agreement, corrected for the level of agreement expected by chance. Values greater than 0.75 indicate excellent agreement, 0.40 to 0.75 fair to good agreement, and values less than 0.40 poor agreement.

### Results

The selection of cases has been reported previously (Gulliford *et al.*, 1991*a*; Gulliford *et al.*, 1991*b*). The Thames Cancer Registry supplied a list of 609 cases with bladder cancer believed to fulfill the entry criteria for the study. For 12 (2%) cases registration was from death certificate only. In 96 cases (16%) neither hospital notes nor radiotherapy records could be retrieved because they were lost or destroyed or the clinician (one surgeon and one radiotherapist) refused access to records. Four patients (0.7%) were found not to have been resident in the South Thames Regions at the time of diagnosis. In seven cases (1%), review of the notes showed that an initial clinical diagnosis of bladder cancer had not been confirmed by subsequent investigation. In a further two cases the biopsy result was normal but the patients were managed clinically as cases of bladder cancer. On review, the year of diagnosis was found not to be 1982 in 24 cases (4%). Further analysis was confined to 466 (77%) cases who were resident in the South Thames regions with a confirmed diagnosis of bladder cancer made in 1982, for whom notes were retrieved for review.

Table I shows agreement for five continuous variables compared for the two sources of data. There was exact agreement for a high proporiton of cases and for discordant

Correspondence: M.C. Gulliford, CAREC, PO Box 164, Port of Spain, Trinidad, WI.
Received 20 July 1992; and in revised form 20 November 1992.

**Table I** Agreement between cancer registry data and data obtained from hospital case notes for continuous variables

| Variable (cases with complete data) | Exact agreement number (%) | No exact agreement number (%) | median (interquartile range) |
|---|---|---|---|
| Date of birth (464) | 432 (93) | 32 (7) | 0 (−32 to 31 days) |
| Date of death (218) | 202 (93) | 16 (7) | 1 (−3 to 5) days |
| Date of first operation (376) | 311 (83) | 65 (17) | −1 (−10 to 3) days |
| Date of first radiotherapy (156) | 138 (88) | 18 (12) | −2 (−18 to 1) days |
| Dose of radiotherapy (146) | 123 (84) | 23 (16) | 14 (1 to 20) Gray |

cases differences were generally of small magnitude. Because the cases were selected according to confirmed date of diagnosis, a valid comparison could not be made for this variable. However, of the 466 cases, date of diagnosis was avialable in both data sets for 430, exact agreement was present in 307 (71%) cases, for discordant cases the median difference was − 1 day (interquartile range − 4 to 7 days).

The level of agreement for classification of histological cell type was also good. 402 cases were recorded as transitional cell carcinoma in the review data and 355 (88%) of these were recorded as transitional cell carcinoma or papillary transitional cell carcinoma in the original data (Table II). High levels of concordance were observed for the smaller numbers of cases in the other categories.

Table III shows the staging information originally recorded at the cancer registry compared with pathological T-stage obtained by reviewing hospital notes: 187 out of 242 (77%) cases recorded as non-invasive (pTa) or micro-invasive (pT1) were recorded as 'local' at the cancer registry. However, 45 out of 93 (48%) of tumours classified as invasive (⩾pT2) were classified as 'local' in the cancer registry data. Thus the categories 'local' and 'local extension' did not adequately distinguish between superficial and invasive bladder tumours. There were 131 (28%) of case notes which gave no pathological indication of tumour stage at review and 74 (16%) for which descriptive information had been missing at registration.

Of 203 cases receiving radiotherapy, 88% were recorded as such at the cancer registry (kappa 0.88 (0.79–0.97)). Of 85 cases receiving chemotherapy, 15% were recorded as such at the registry (kappa 0.41 (0.32–0.49)).

### Discussion

The Thames Cancer Registry employs specially trained peripatetic clerks to complete cancer registration forms. This report suggests that the data abstracted by these clerks generally showed a high level of agreement with data independently abstracted from hospital records for another study. This finding confirms observations on the reliability of abstraction of data made in the United States (Horwitz & Yu, 1984). In particular it was reassuring that the dates of initial treatment and death, which may be used in analysis of patient survival, appeared to be reliably recorded in the cancer registry records for the majority of cases.

The selection of cases for this study was not unbiased (Gulliford *et al.*, 1991*a*). In addition to a high proportion of cases for whom case notes could not be retrieved, a number of cases were excluded. These included seven cases in whom a diagnosis of bladder cancer had not been confirmed. Usually an initial clinical diagnosis of bladder cancer had not been supported by subsequent investigation, either because the tumour was found to originate in another organ or because a non-neoplastic pathology was identified. In 24 cases the date of diagnosis was found not to have been 1982. In most of these cases a diagnosis of superficial bladder cancer (papilloma) had been made a number of years

**Table II** Histological classification of tumour ascertained from hospital records and from the cancer registry

| Hospital records | Cancer registry (ICD-O code) | | |
|---|---|---|---|
| Transitional cell ca (TCC) N = 402 | M81203 TCC NOS | 246 | |
| | M81303 papillary TCC | | 109 |
| | M80103 carcinoma NOS | | 18 |
| | M80503 papillary carcinoma NOS | 13 | |
| | Eight other codes | | 16 |
| Squamous cell ca (SCC) N = 7 | M80703 SCC NOS | | 6 |
| | M80713 SCC Keratinising type | | 1 |
| Anaplastic ca N = 7 | M80103 carcinoma NOS | | 5 |
| | M81203 TCC NOS | | 1 |
| | M81403 Adenocarcinomas | | 1 |
| Carcinoma in situ (CIS) N = 6 | M80102 CIS NOS | | 4 |
| | M81202 TCC in situ | | 1 |
| | M81303 papillary TCC | | 1 |
| Normal biopsy N = 2 | M80001 neoplasm, NOS | | 1 |
| | M81201 urothelial papilloma | | 1 |
| Adenocarcinoma (3) | M81403 adenocarcinoma NOS | | 3 |
| Spindle cell ca (1) | M80323 spindle cell carcinoma | | 1 |
| Malignant fibrous histiocytoma (1) | M80323 spindle cell carcinoma | | 1 |
| Other uncertain (1) | M82513 alveolar adenocarcima | | 1 |
| Not known (N = 36) | | | |

NOS = Not otherwise specified.

**Table III** Comparison of measures of tumour invasion recorded from hospital records and cancer registry data. (Figures are frequencies (percentage of row total))

| *Hospital records (Review)* | | *Thames Cancer Registry (Original)* | | | |
|---|---|---|---|---|---|
| *pT category* | *Number of patients* | *Local* | *Local extension* | *Nodal or distant metastases* | *Not known* |
| pTa | 154 | 119 (77) | 8 (5) | 3 (2) | 24 |
| pT1 | 88 | 68 (77) | 10 (11) | 0 (0) | 10 |
| ⩾pT2 | 93 | 45 (48) | 20 (22) | 6 (6) | 22 |
| Not known | 131 | 97 | 10 | 6 | 18 |
| Total | 466 | 329 | 48 | 15 | 74 |

previously, at a time when the rules for registration of these neoplasms by the registry were different. These cases were not analysed further but their exclusion meant that the reliability of date of diagnosis could not be fully evaluated.

This study was confined to tumours at a single site so it was not possible to analyse the reliability of recording of topographic information but it is relevant to note that for one per cent of the original sample, a diagnosis of bladder cancer was not confirmed on review. The original coding of tumour morphology was consistent with that independently obtained by reviewing clinical records.

It was clear that the recording of tumour stage presented particular problems. In each data set there was a high proportion of clinical records from which the tumour stage could not be clearly identified. The relatively high proportion of hospital records which did not include mention of the clinical stage has also been noted in an American study (Fiegl *et al.*, 1988). In our study we found that the categories of the TNM classification were rarely explicitly mentioned either in clinical records or pathology reports, although the latter often included a written statement of the information required for classification (unpublished observations). The poor quality of staging information contained in clinical records was one of the reasons why the cancer registry introduced a simplified staging system in 1981. However, the categories of this simplified system did not allow separation of superficial from invasive bladder tumours. Thus the recording of staging information by clinicians and pathologists needs to be improved before their records can be used as a reliable source of information concerning tumour stage.

The cancer registry aims to collect data concerning initial treatment within the first 6 months after diagnosis. The present results show that this approach results in an incomplete record of the use of these treatment modalities when compared with clinical records examined a number of years later.

Cancer registry data showed good agreement with information independently abstracted from patients' clinical records. Recording of staging information in the clinical setting appeared to be unsatisfactory because of the high proportion of cases for which tumour stage was not mentioned and the infrequent explicit use of the TNM classification. The quality of staging data available to registries needs to be improved. Increasing involvement of clinicians and pathologists in registration might provide one method of achieving this.

We thank the clinicians who allowed us to examine their patients' notes and Professor W.W. Holland for his support. The authors were supported by the Wellcome Trust and the Department of Health.

### References

CANCER RESEARCH CAMPAIGN (1982). *Trends in cancer survival in Great Britain*. Cases registered between 1960 and 1974. Cancer Research Campaign: London.

FIEGL, P., GLAEFCKE, G., FORD, L., DIEHR, P. & CHU, J. (1988). Studying patterns of cancer care: how useful is the medical record? *Am. J. Public Health*, **78**, 526–533.

FLEISS, J.L. (1981). *Statistical Methods for Rates and Proportions*. 2nd edition. pp. 211–236. Wiley: New York.

GILLIS, C.R., HOLE, D.J., STILL, R.M., DAVIS, J. & KAYE, S.B. (1991). Medical audit, cancer registration and survival in ovarian cancer. *Lancet*, **i**, 611–612.

GULLIFORD, M.C., PETRUCKEVITCH, A. & BURNEY, P.G.J. (1981*a*). Hospital case notes and medical audit: evaluation of non-response. *Br. Med. J.*, **302**, 1128–1129.

GULLIFORD, M.C., PETRUCKEVITCH, A. & BURNEY, P.G.J. (1991*b*). Survival with bladder cancer: evaluation of delays in treatment, type of surgeon and modality of treatment. *Br. Med. J.*, **303**, 437–440.

HORWITZ, R.I. & YU, E.C. (1984). Assessing the reliability of epidemiologic data obtained from medical records. *J. Chron. Dis.*, **37**, 825–831.

NWENE, U. & SMITH, A. (1982). Assessing completeness of cancer registration in the North Western region of England by a method of independent comparison. *Br. J. Cancer*, **46**, 635–639.

OFFICE OF POPULATIONS CENSUSES AND SURVEYS (1985). *Cancer statistics*. Series MB1. HMSO: London.

OFFICE OF POPULATION CENSUES AND SURVEYS (1990). *Review of the national cancer registration system*. Series MB1 no. 17. HMSO: London.

WATERHOUSE, J., MUIR, C., SHANMUGARATNAM, K. & POWELL, J. (1982). Cancer incidence in five continents, volume IV. International Agency for Research on Cancer: Lyon.

WORLD HEALTH ORGANISATION (1976). International Classification of Diseases for Oncology. First edition. WHO: Geneva.